# Natural autophagy blockers, dauricine (DAC) and daurisoline (DAS), sensitize cancer cells to camptothecin-induced toxicity

**DOI:** 10.18632/oncotarget.20767

**Published:** 2017-09-08

**Authors:** Ming-Yue Wu, Sheng-Fang Wang, Cui-Zan Cai, Jie-Qiong Tan, Min Li, Jin-Jian Lu, Xiu-Ping Chen, Yi-Tao Wang, Wei Zheng, Jia-Hong Lu

**Affiliations:** ^1^ State Key Laboratory of Quality Research in Chinese Medicine, Institute of Chinese Medical Sciences, University of Macau, Macau SAR, China; ^2^ State Key Laboratory of Medical Genetics, Xiangya Medical School, Central South University, Changsha, Hunan, China; ^3^ Mr. and Mrs. Ko Chi Ming Centre for Parkinson's Disease Research, School of Chinese Medicine, Hong Kong Baptist University, Hong Kong SAR, China; ^4^ Department of Thyroid and Breast Surgery, The Third People's Hospital of Shenzhen, Shenzhen, Guangdong, China

**Keywords:** dauricine, daurisoline, autophagy blocker, cancer cells, cell toxicity

## Abstract

Autophagy is a cellular bulk degradation pathway implicated in various diseases. Inhibition of autophagy has been regarded as a new therapeutic strategy for cancer treatment, especially in combination with chemotherapy. In our study, we identified two natural compounds, dauricine (DAC) and daurisoline (DAS), as two potent autophagy blockers through a high-content screening. DAC and DAS are alkaloids isolated from traditional Chinese medicine *Rhizoma Menispermi*. We systematically examined the effects of DAC and DAS on autophagy function in HeLa cells and found that DAC and DAS induced massive formation of autophagic vacuoles and lipidation of LC3. The accumulation of autophagic vacuoles and LC3 lipidation are due to blockage of autophagosome maturation as evidenced by interrupted colocalization of autophagsosome and lysosome, increased GFP-LC3/RFP-LC3 ratio and accumulation of autophagic substrate p62. Moreover, DAC and DAS impaired lysosomal function, as indicated by reduced lysosomal protease activity and increased lysosomal pH values. Importantly, we showed that DAC and DAS strongly inhibited the lysosome V-type ATPase activity. For the therapeutic potential, we found that DAC and DAS blocked the campothecin (CPT)-induced protective autophagy in HeLa cells, and dramatically sensitized the multiple cancer cells to CPT-induced cell death. In conclusion, our result shows that DAC and DAS are autophagy inhibitors which inhibit the lysosomal degradation of auophagic vacuoles, and sensitize the CPT-induced cancer cell death. The study implies the therapeutic potential of DAC and DAS in the treatment of cancers in combination of chemotherapy by inhibiting autophagy.

## INTRODUCTION

Dauricine (C_38_H_44_N_2_O_6_) (DAC) and daurisoline (C_37_H_42_N_2_O_6_) (DAS) are two isoquinoline alkaloids isolated from Chinese herbal medicine *Rhizoma Menispermi* which has been used in the decoction to treat various diseases including cancers. DAC and DAS have been reported to show potential pharmacological efficacy in many diseases, such as focal ischemia/reperfusion injury [1, 2], arrhythmia [3, 4], and platelet aggregation [5, 6] via suppressing NF-κB pathway [7], antagonizing potassium and calcium channel [8, 9], and inhibiting thromboxane A_2_ (TXA_2_) function [10]. Recent years, DAC has been reported to inhibit the proliferation of tumor cells, such as urinary tract tumor cell [11] and colon cell [12], which revealed a new potential therapeutic direction for the two compounds. However, limited evidences could elucidate the mechanism that involved in tumor cell growth inhibition.

Autophagy is a conserved cellular degradation pathway, which is responsible for the clearance of the damaged organelles, superfluous proteins, and pathogens via autophagosome engulfment and lysosomal degradation. A growing body of evidence has linked autophagy to diverse diseases [13] including cancer. Autophagy is a double-edged sword for cancer. On the one hand, autophagy defects have been associated with increased tumorigenesis [14–17]. On the other hand, autophagy serves as a survival mechanism for tumor cells under metabolic stresses [18–20]. In recent years, increasing evidence strongly suggests that autophagy is fundamentally elevated in cancer tissue regardless of Akt/mTOR signal activation [21–23], indicating that autophagy has been induced mostly as a protective mechanism in cancer cells. Therefore, autophagy inhibitors have been considered as potential therapeutic strategy for cancer, and several autophagy inhibitors are under clinical trials either as monotherapy or in combination with chemotherapies [24–26]. Besides the well-known autophagy inhibitors, other candidates from natural compounds may be found to facilitate cancer therapy.

DAC and DAS have been reported as the small-molecule enhancers of autophagy to induce autophagic cell death in certain cancer cells [27]. However, our study reports DAC and DAS as potent autophagy blockers rather than inducers. We showed that these two compounds increased the accumulation of autophagic vacuoles by attenuating the fusion of the autophagosome and lysosome, and impairing lysosomal function. Camptothecin (CPT) is an anti-cancer drug isolated from Camptotheca acuminata and protective autophagy activation has been observed when cells were treated with CPT [28]. Our data shows that DAC and DAS dramatically blocked CPT-induced protective autophagy and sensitized HeLa cell, HCT 116, and A549 cells to CPT-induced cell toxicity, implying the potential use of DAC and DAS in the chemotherapy where protective autophagy are induced.

## RESULTS

### Daurocine (DAC) and daurisoline (DAS) are autophagy modulators which increased autophagic vacuoles (AVs) in HeLa cell

**Figure 1:**
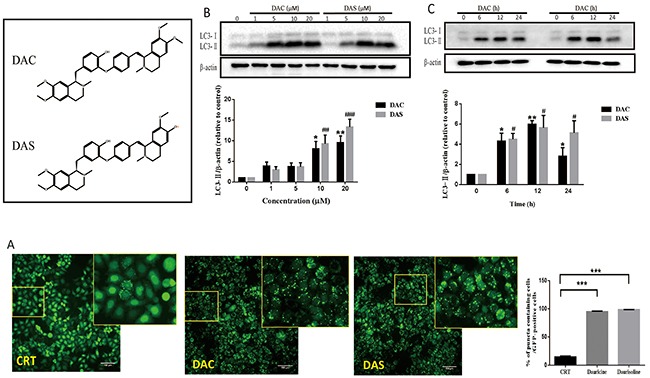
Autophagic vacuoles are induced by DAC and DAS treatment **(A)** GFP-LC3 puncta increase in DAC- and DAS-treated HeLa cells. The images of GFP-LC3-expressed HeLa cells were detected under InCell 2000 system after treatment of 10 μM DAC and DAS or vehicle (DMSO) for 24 h. Bars, 100μm. **(B)** DAC and DAS increased LC3 in a dose dependent manner. The LC3 expression in HeLa cells after treatment of different concentrations of DAC and DAS for 24 h; The relative intensity of LC3-IIexpression was calculated via Image J. (*P, ^#^P < 0.05, **P < 0.01, ^###^P < 0.001 *versus* CRT). **(C)** LC3-II expression after treatment of DAC and DAS at different time points. The LC3 expression in HeLa cells at different time points after treatment with 10 μM DAC and DAS were analyzed via western blotting. The relative intensity of LC3-II expression was calculated via Image J. (*P, ^#^P < 0.05, **P < 0.01 *versus* CRT). Error bars (mean±SEM). One way ANOVA with Turkey as post hoc tests.

To identify the effects of DAC and DAS on autophagic AVs, we analyzed fluorescent images of stable microtubule-associated protein light chain 3 (LC3)-expressing HeLa cell after treatment of compounds including DAC and DAS via *In Cell* 2000 (GE Healthcare). GFP-LC3 has been widely used as a autophagosome marker to indicate the formation of autophagosome in cells [30]. The high content screening identified dauricine (DAC) and daurisoline (DAS) as autophagy modulators which dramatically increased AVs. As shown in Figure 1A, treatment of DAC and DAS at 10 μM increased LC3-positive puncta dramatically in GFP-LC3 HeLa cell. Furthermore, DAC and DAS dose-dependently increased the formation of LC3-II (lipidation from of LC3) from 1 to 20 μM (Figure 1B), and also induced LC3 accumulation up to 24 h (Figure 1C).

### DAC and DAS inhibit the autophagic degradation in HeLa and MEF cells

**Figure 2:**
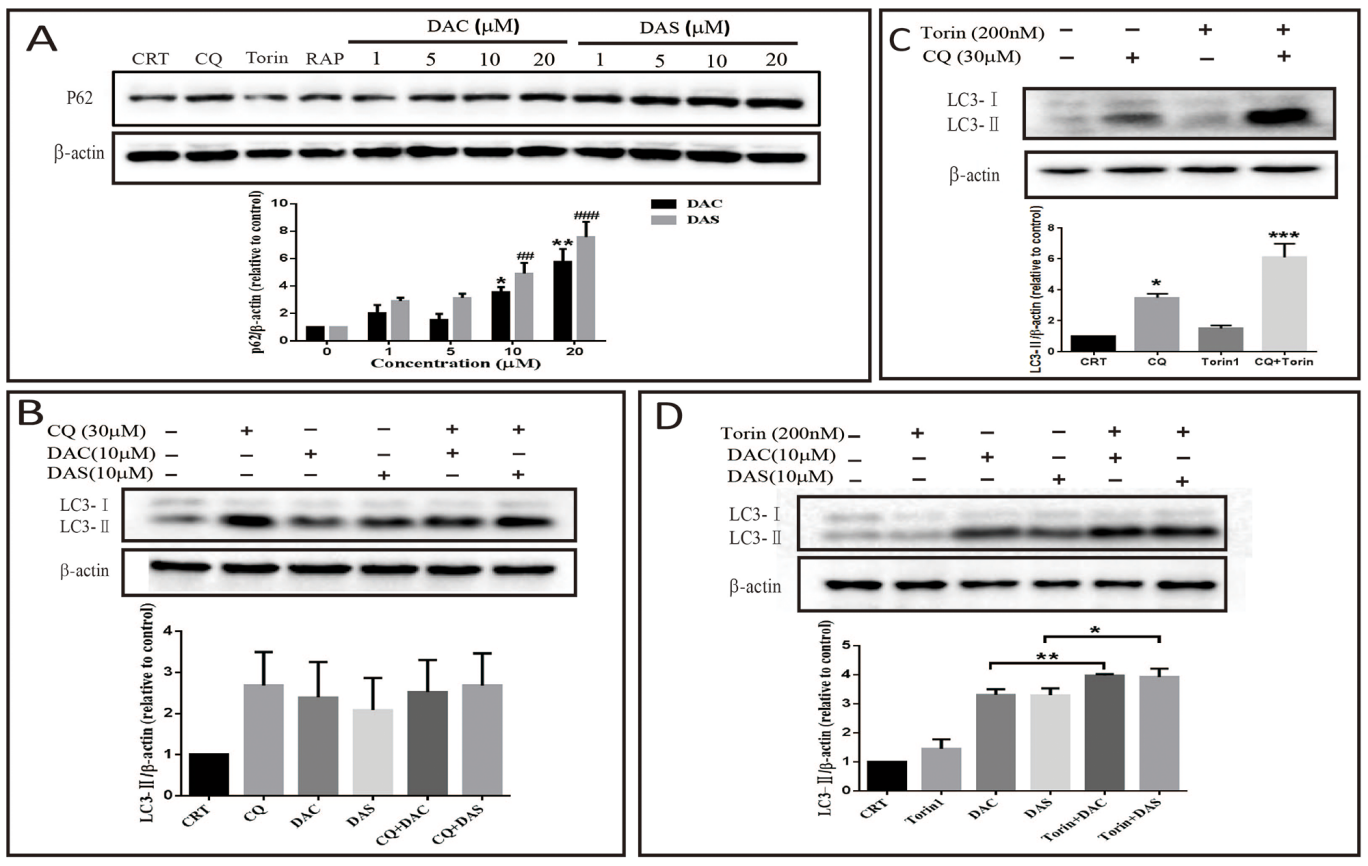
DAC and DAS inhibit autophagic degradation in HeLa and Mouse Embryo Fibroblast (MEF) cells **(A)** HeLa cells were treated with different concentrations of DAC and DAS for 24 h, and the P62 expression were measured by western blotting (*P < 0.05, ^##^P < 0.01, ^###^P < 0.001 *versus* control group). **(B)** Western blotting results of LC3-II in the DAC- or DAS-treated HeLa cell with or without CQ (30 μM). **(C)** HeLa cells were treated with DMSO or 30 μM CQ for 6 h in the presence or absence of 200 nM Torin for last 3 h (*P < 0.05, ***P < 0.001 *versus* control group). **(D)** HeLa cells were treated with DMSO, 10 μM DAC or 10 μM DAS for 6 h with or without treatment with 200 nM Torin for last 3 h. MEF cells were treated with DAC and DAS in different concentrations for 24 h, and the expression of P62 and LC3 were measured by western blotting. Relative intensity of p62 and LC3-II was calculated by Image J (*P < 0.05, **P < 0.01). Error bars are mean±SEM. One way ANOVA with Turkey as post hoc tests.

Autophagic process can be separated into several steps: initiation, elongation, maturation, and degradation. The accumulated AVs may be resulted from induced initiation or blockage of degradation. To dissect the process of DAC and DAS-induced AVs accumulation, expression levels of p62 and LC3 were detected in different conditions. Rapamycin (RAP) and Torin are two well-known autophagy enhancers via inhibiting mTOR kinase [31]. As shown in Figure 2A, autophagic substrate p62 expressions were increased significantly after DAC and DAS treatment, but decreased in the cells treated with RAP and Torin. To monitor the autophagy influx, lysosome degradation blockers ie. Chloroquine (CQ) are used to block the degradation of LC3. Figure 2B shows that treatment of DAC and DAS hardly increase LC3-II expression in the presence of CQ when compared to CQ treatment alone. In contrary, as Figure 2C showed that classic autophagy inducer Torin1 further increased the LC3-II expression in the presence of CQ when compared with CQ treatment alone. Moreover, as Figure 2D shown, Torin increased the LC3-II expression in the presence of DAC and DAS when compared with DAC and DAS treatment alone, respectively. To investigate the effects on the normal cell line, we determined the p62 and LC3-II expression on the Mouse Embryo Fibroblast (MEF) cells. As Supplementary Figure 1 shown, DAC and DAS obviously enhanced the p62 and LC3-II expressions on MEF cell. Taken together, the data indicates that DAC and DAS inhibit the autophagic degradation in HeLa and MEF cells.

### DAC and DAS inhibit the fusion of autophagosome and lysosome in HeLa cell

**Figure 3:**
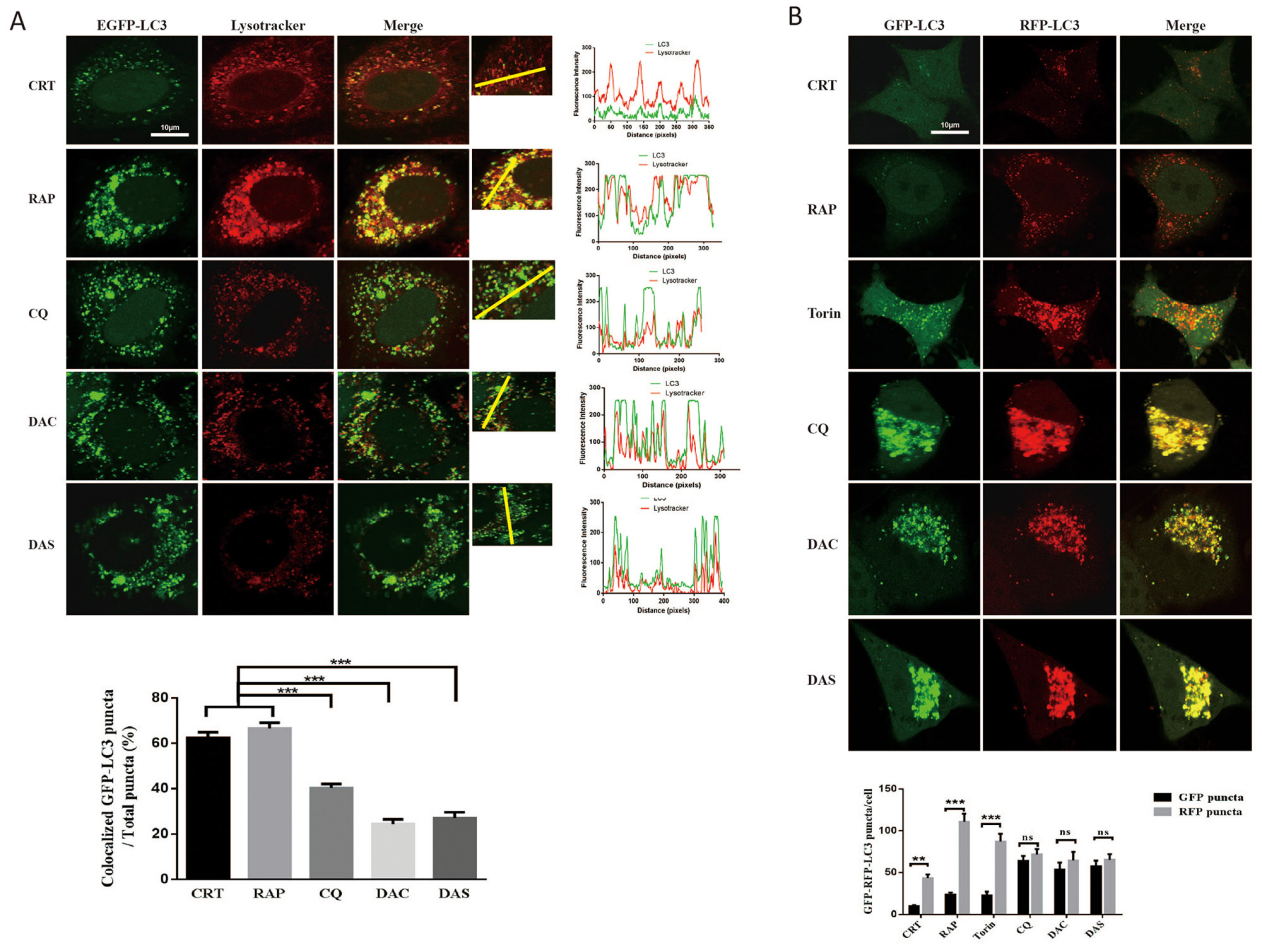
DAC and DAS inhibit the fusion of autophagosome and lysosome in HeLa cell **(A)** Co-localization analysis of EGFP-LC3 and Lysotracker. GFP-LC3 HeLa cells were treated with 10 μM DAC, 10 μM DAS, 30 μM CQ, 1 μM RAP, or DMSO for 24 h. The fluorescence images of LC3 and lysotracker were scanned with different channels via a confocal microscopy. The LC3 puncta that co-localizated with lysotracker were counted from at least 20 cells. Bar, 10μm. **(B)** Analysis of GFP-RFP-LC3 fluorescent signals in HeLa cells. HeLa cells were transiently transfected with GFP-RFP-LC3 plasmid, and treated with 10 μM DAC, 10 μM DAS, 30 μM CQ, 1 μM RAP, 200 nM Torin or DMSO for 24 h. The fluorescence images of LC3 were scanned with different channels under a confocal microscopy. Bar, 10μm. GFP or RFP puncta were counted at least in 20 cells.**P < 0.01, ***P < 0.001. Error bars are mean±SEM. One way ANOVA with Turkey as post hoc tests.

Autophagosome clearance depends on its fusion with lysosome. In order to elucidate whether DAC and DAS treatment also inhibit the fusion process, we analyzed the co-localization of autophasosome and lysosome markers. Firstly, we stained the lysosome by lysotracker on GFP-LC3 HeLa cell, and measured the colocalization of GFP-LC3 and lysotracker as shown in Figure 3A. The GFP-LC3 puncta levels increased significantly after treatment of DAC and DAS, whereas, their colocalization with lysotracker was not increased. Furthermore, we also transfected HeLa cell with RFP-GFP-LC3 construct. Because GFP fluorescence is easily quenched in an acidic environment, the red-only puncta after merging fluorescence indicate the autophasome maturation. On the contrary, the blockage of fusion results in yellow puncta after merging images. In Figure 3B, after treatment with DAC and DAS, yellow puncta were markedly increased after merging fluorescent images, indicating that both DAC and DAS treatment block the maturation of autolysosome. Taken together, our results suggest that both DAC and DAS inhibit the autophagy maturation process by interrupting the fusion of autophasome and lysosome.

### DAC and DAS treatment impaired lysosomal function and lysosome acidity

**Figure 4:**
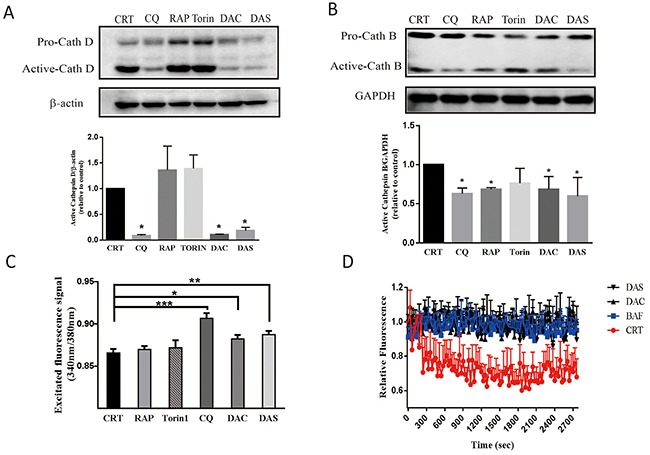
DAC and DAS treatment impaired lysosomal function in HeLa cell **(A)** and **(B)** Treatment of DAC and DAS decreased the expressions of active-cathepsin D and active-cathepsin B. The cathepsin D, and cathepsin B expression in HeLa cell were detected by western blotting after treatment with 10 μM DAC, 10 μM DAS, 200 nM Torin, 30 μM CQ, 1 μM RAP or DMSO for 12 h. Relative intensity of cathepsin D expression were calculated by image J. **(C)** DAC and DAS increase the ratio of excited fluorescence signal at 340 nm and 380 nm. HeLa cell were stain with LysoSensor Yellow/Blue DND-160 after treatment of 10 μM DAC, 10 μM DAS, 30 μM CQ, 1 μM RAP or DMSO for 24 h. The excited fluorescence signal at 535 nm were measured by 96-well plate reader. **(D)** Inhibitory effect of DAC and DAS on v-ATPase activity. V-ATPase-mediated acidification of fractionated lysosomes with dextran-OG514 was started by addition of 5mM ATP + MgCl_2_, in the absence of DMSO, 50 nM BAF, 20 μM DAC or 20 μM DAS. *P < 0.05, **P < 0.01, ***P < 0.001. Error bars are mean±SEM. One way ANOVA with Turkey as post hoc tests.

Lysosomal protease activity and its acidic environment is critical for the lysosome function. Cathepsin D and cathepsin B are two major proteases in lysosome. The premature procathepsin exists in cytoplasm and active form exits in endosome and lysosome [32]. Acidic pH in (4.5-5) the lumina of lysosome contributes to an effective trafficking and proteolytic activation of lysosomal proteases. To understand whether the two compounds impair the lysosomal function to block the autophagosome maturation, we examined the active cathepsin D, B levels and lysosome pH value. As Figure 4A and 4B shown, active cathepsin D and cathepsin B levels decreased significantly after treatment of DAC and DAS. Lysosensor Yellow/Blue DND-160 can be used to monitor the lysosomal pH by calculating the ratio of acquired fluorescent signals emitted at 535nm which were excited at 340nm and 380nm, respectively. As shown in Figure 4C, the lysosome pH values after DAC and DAS treatment were significantly higher than CRT, RAP and Torin. CQ, a well-known agent to inhibit acidification, was used as positive control. Furthermore, to figure out how DAC and DAS affect the lysosome function and acidity, we detected the activity of V-type ATPase, the proton pump to produce a proton gradient on organelles, by measuring the acidification of fractionated lysosomes with dextran-OG514. Bafilomycin A1 (BAF) is a lysosome V-type ATPase activity inhibitor and used as positive control here. As Figure 4D shown, similar to the treatment of BAF, pretreatment of DAC and DAS inhibited the lysosome acidifying process after addition of ATP and MgCl_2_ when compared to control. Thus, the data demonstrate that DAC and DAS impair lysosomal function and lysosomal acidification, via inhibiting the lysosome V-type ATPase acitivity in DAC and DAS treated cells.

### DAC and DAS blocked CPT-induced protective autophagy

**Figure 5:**
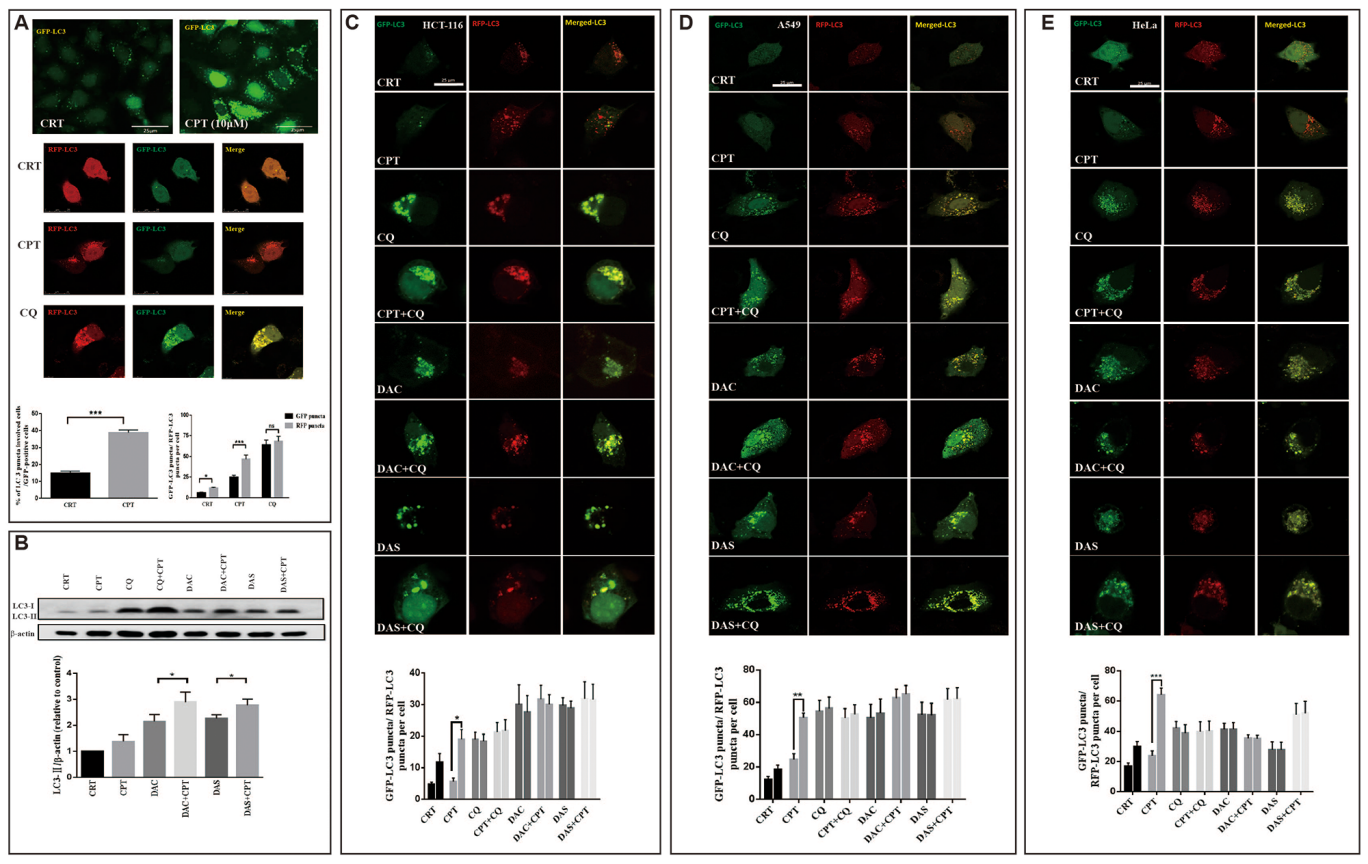
DAC and DAS blocked CPT-induced autophagy **(A)** CPT induces autophagy in HeLa cells. HeLa-GFP-LC3 cells were treated with DMSO or 10 μM CPT for 24 h, or HeLa cells with GFP-RFP-LC3 transfection were treated with DMSO, 20 μM CPT or 20 μM CQ for 24 h, and the fluorescence images were determined by fluorescence microscopy. Bars, 25 μm. **(B)** DAC and DAS inhibit CPT-induced autophagy. HeLa cells were treated with DMSO, 20 μM DAC, 20 μM DAS, 20 μM CPT alone or combined with DAC or DAS for 6h, the whole cell lysates were subjected to western blot against LC3. **(C-E)** DAC and DAS blocked the CPT induced autophagy. HCT-116 cell, A549 cell and HeLa cell with GFP-RFP-LC3 transfection were treated with DMSO, 20 μM CPT, 30 μM CQ, 20 μM DAC, 20 μM DAS or DAC and DAS in the presence of CPT for 12 h. The fluorescence images were obtained by Confocal microscopy. Bars, 25 μm. *P<0.05, **P<0.01, ***P<0.001. Error bars (mean±SEM). One-way ANOVA with Turkey as post hoc tests.

Autophagy induction is a survival mechanism for tumor cells under nutrition deprivation and chemotherapy conditions. Camptothecin (CPT) is a natural anti-cancer drug used in the multiple cancer treatment and has been shown to induce protective autophagy in several cancer cells [33, 34]. To investigate whether DAC or DAS can block the CPT-induced protective autophagy, we detected the CPT-induced autophagy and determined the autophagy blockage function of DAC and DAS after CPT treatment. As shown in Figure 5A, CPT significantly increased LC3 puncta in HeLa cells stably-overexpressing GFP-LC3 and enhanced the maturation of autolysosome, evidenced by an increase in the RFP-LC3-only puncta (Figure 5A), indicating that CPT enhanced autophagy in HeLa cell. In Figure 5B, CPT-induced LC3-II were further enhanced in the presence of DAC and DAS when compared with DAC and DAS treatment alone, indicating a blockage of CPT-induced autophagy by DAC and DAS. To investigate the blockage of DAC and DAS on CPT-induced autophagy, we used HeLa cell, HCT116 cell, and A549 cells which were transfected with RFP-GFP-LC3 to determine the maturation of autolysosome. As in Figure 5C-5E shown, DAC and DAS obviously decreased the RFP-LC3-only puncta when compared to CPT-only treated cells, revealing the blocked autolysosome maturation. The above data indicated DAC and DAS inhibit the CPT-induced autophagy in different cancer cell lines.

### DAC and DAS aggravated CPT-induced toxicity in multiple cancer cells

**Figure 6:**
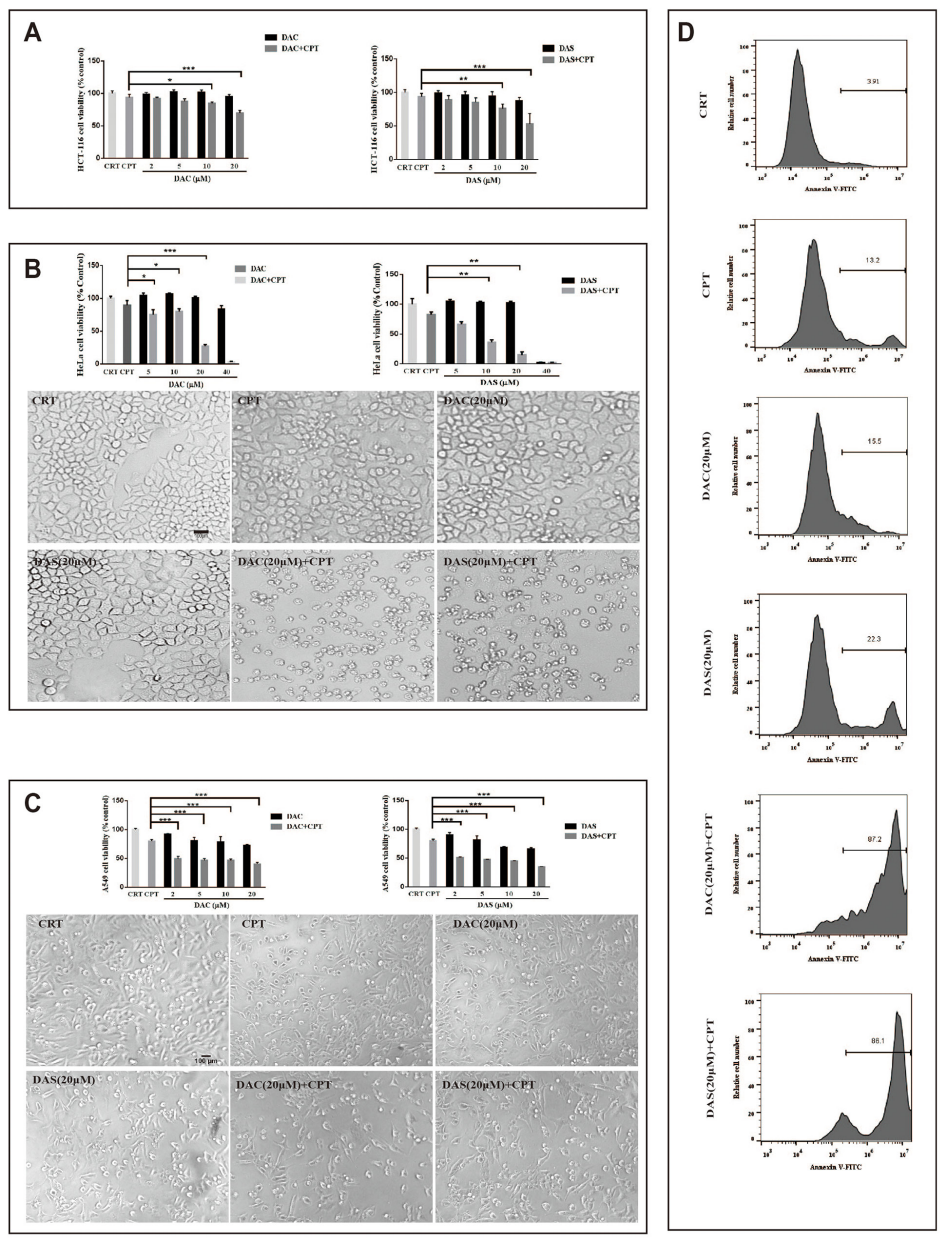
DAC and DAS aggravated CPT-induced toxicity in multiple cancer cells **(A-C)** Cells were treated with DMSO, CPT and different concentrations of DAC and DAS alone or combined with CPT for 48 h. Cell morphology images were obtained under microscopy. **(D)** HeLa cells were treated with DMSO, CPT and DAC and DAS alone or combined with CPT for 48 h. Annexin V staining were determined by flow cytometry. *P<0.05, **P<0.01, ***P<0.001. Error bars are mean±SEM. One-way ANOVA with Turkey as post hoc tests.

In recent years, autophagy inhibition has been regarded as a new strategy for cancer therapy via blocking the chemotherapy induced-protective autophagy. To test whether DAC and DAS sensitize CPT-induced cancer cell death via autophagy inhibition, the CPT-induced cell viability inhibition and morphological changes were determined in the presence or absence of DAC and DAS in multiple cancer cell lines: HeLa cells, HCT 116 cells and A549 cells (IC_50_ of DAC and DAS in different cancer cells have been shown in the Supplementary Table 1). According to the preliminary pilot experiment, 48 h is a better period for DAC and DAS to display their function of inhibiting protective autophagy induced by CPT. The data in Figure 6A-6C showed that combined treatment of CPT with DAC or DAS reduced cell viability dramatically and caused typical toxic morphology compared with CPT, DAC or DAS treatment alone in HeLa cells, HCT 116 cells and A 549 cells. In addition, the flow cytometry result of Annexin-V staining in Figure 6D displayed the dramatically increased proportions of Annexin V positive cells in the presence of DAC or DAS when compared to the only CPT-treated HeLa cells. Collectively, these data demonstrate that DAC and DAS are able to sensitize the CPT-induced cancer cell death probably through the suppression of protective autophagy.

## DISCUSSION

Previous study has reported the enhanced LC3 expression in different cell types that treated with DAC [27]. However, the accumulation of AVs is not a sufficient evidence to indicate the enhancement of autophagy, it could also be resulted from the blockage of autophagic degradation. For example, Tetrandrine (Tet) and Matrine are two kinds of traditional Chinese medicine-derived compouds, and both of them have been reported as autophagy inducers in different cell lines [25, 35, 36]. However, recent studies demonstrated that both of them blocked the autophagic flux by lysosomal deacidification [37] or inhibiting lysosomal proteases [38]. Another typical example is thapsigargin, an endoplasmic reticulum (ER) stressor which had been report as autophagy inducer, actually blocks autophagy by inhibiting autophagsome-lysosome fusion [39]. Furthermore, a recent study using autophagy maturation probe systematically analyzed the effects of commonly reported autophagy modulators and found that a big portion of reported autophagy inducers are actually blockers [40]. The contrary reports suggest that it worth trying to re-examine chemical autophagy modulators in multiple systems and experimental conditions to avoid condition-specific effects, or even wrong interpretation of data. In this study, we revealed that the accumulation of AVs in DAC- and DAS-treated HeLa cells were due to the inhibition of autophagic degradation, rather than inducing AVs formation. We report that DAC and DAS are autophagy inhibitors via suppressing lysosomal functions. DAC and DAS treatment alter the lysosomal homeostasis via elevating the lysosomal pH, resulting the suppressed enzyme activities in lysosome, such as cathepsin D and cathepsin B. Afterwards, fusion of autophagsome and lysosome, and autophagic degradation are failed by the disrupted lysosomal function.

Mechanistically, autophagosome maturation inhibitors impair the autophagosome and lysosome fusion by two major ways: i, inhibiting the V-type ATPase activity which maintain lysosomal pH gradient (BAF) [41], ii, diffusing into the lysosome in the monoprotonated form as a lysosomotropic weak base, resulting elevation of lysosomal pH (CQ) [42]. In our study, we found DAC and DAS both show ability to inhibit V-type ATPase activity on lysosome, which may be the potential mechanism of lysosomal deacidification and resulting the autophagosome and lysosome fusion problem.

Based on the previous studies of DAC and DAS, these two kinds of compounds have been reported as L-type calcium current inhibitor [43, 44]. In recent years, more and more papers have revealed the potential connections of calcium signaling and autophagy [45, 46]. Diego L. et al. prove that lysosomal calcium signaling regulates autophagy through calcineurin and TEFB [47]. It would be interesting to explore the potential connection between calcium channel inhibition, V-type ATPase inhibition and the fusion of autophagsosome and lysosome in the future.

Autophagy has been continuously reported as a prosurvival and resistance mechanism against chemotherapy treatment [48–50]. Emerging evidence has demonstrated that inhibition of protective autophagy promoted apoptosis and attenuated cancer growth through sensitizing tumor cells to chemotherapy. Especially, Chloroquin (CQ), a canonic autophagy inhibitor, has been combined with a wide range of anti-cancer drugs to enhance the cytotoxicity in multiple cell models [51–53], and its combination with chemotherapy are under investigation in a list of clinical trials [54, 55]. Although the efficiency of CQ for cancer therapy is promising, the side effect of CQ has to be taken into consideration. For example, mice poorly tolerate CQ and do not improve overall survival after long-term chronic administration [56]. Therefore, there is a need to discover other candidate of autophagy inhibitors with better performance. CPT is a well-known anti-cancer drug, and has been proved to induce the protective autophagy in certain cancer cells. In our study, we use the DAC and DAS as autophagy inhibitors to block the CPT-induced protective autophagy in cancer cells, so that to sensitize the cancer cells to the CPT-induced apoptosis. Long period chemotherapy-caused drug resistance is the major reason of chemotherapy fail. It is important to find ways to improve the efficiency and prevent the drug-resistance of anti-cancer drugs. Our result suggests that DAC and DAS have the potential for the synergetic treatment for cancer therapy as a way to increase the efficiency of chemotherapy by inhibiting autophagy.

## MATERIALS AND METHODS

### Chemicals

Dauricine and daurisoline (purity >98%) were purchased from Must Bio-technology Co., Ltd (Chengdu), China. ATP, Rapamycin (RAP), Torin, Bafilomycin A1 (BAF) and Chloroquine disphosphate (CQ) were obtained from Sigma-Aldrich (St Louis, MO). Dx-OG514 was purchased from Invitrogen (Carlsbad, CA). Antibodies against β-actin and LC3 were purchased from Cell Signaling Technology (Danvers, MA). The p62 antibody was obtained from Progen (Heidelberg, Germany). Antibody against cathepsin D was purchased from Santa Cruz Biotechnology (Santa Cruz, CA).

### Cell culture

HeLa cells stably expressing EGFP-LC3 were established by G418 selection. Cells were cultured in Dulbecco's modified Eagle's medium (DMEM) supplemented with 10% fetal bovine serum and 1% penicillin and streptomycin. 200ng/ml of G418 was added to maintain the selection. All cultures were incubated at 37°C in a humidified atmosphere containing 5% CO_2_.

### Cell proliferation assay

Cell proliferation was determined by the 3-(4,5-dimethylthiazol-2-yl)-2,5-diphenyltetrazolium bromide (MTT) assay. HeLa cells were seeded at 7000 cells per well in 96-well plates in DMEM (1% serum). After cells were treated with different compounds for indicated times, 20 μL of MTT (2.5 mg/ml in PBS) was added to each well. The plates were incubated for an additional 4 h at 37°C. Then the purple-blue MTT formazan precipitate was dissolved in 100 μL DMSO. The cell viability of HeLa cell was evaluated by measuring optical density at 572 nm with a microplate reader.

### Transfection

The mcherry-GFP-LC3 plasmid was kindly provided by Dr. Terje Johansen (University of Tromsø, Norway). HeLa cells were transfected with plasmid for 24 h by Lipo3000 transfection kit (Invitrogen). 24 h after transfection, HeLa cells were treated with compounds at arranged time and concentration.

### Fluorescent Images

#### GFP-LC3 fluorescence images

Stable EGFP-LC3 HeLa cells were seeded on 96-well plate at a density of 7000/well, and treated with different compounds at 10 μM or 50 μM for 24 h. Then, the cells were fixed in 4% paraformaldehyde in phosphate-buffered saline (PBS) at room temperature for 10 min. After wash for two times, the fluorescent signals were monitored via *In Cell 2000* system (GE Healthcare). To quantify autophagy, cells with obvious GFP-LC3 puncta formation were counted and divided by the total number of GFP-positive cells. A minimum of 600 cells from randomly selected fields were counted.

#### Mcherry-GFP-LC3 and lysotracker fluorescence images

Stable EGFP-LC3 HeLa cells and transiently mcherry-GFP-LC3-expressed HeLa cells were seeded in 24-well plate with glass coverslips in each well and were incubated with vehicle, RAP, CQ or 10 μM DAC and DAS for 24 h. Then, the cells were stained with 50 nM Lysotraker Red DND-99 (Molecular Probes, Eugene, OR) in the pre-warmed DMEM without serum for 30 min at 37°C. After fixation with 4% paraformaldehyde, cells were examined under a confocal fluorescence imaging microscope (Leica TCS SP8; Leica Microsystems).

### Western blot

Cells were lysed with RIPA buffer (50 mM Tris-HCl, 1% NP40, 0.35% DOC, 150 mM NaCl, 1 mM EDTA, 1 mM EGTA, supplemented with protease and phosphatase inhibitor cocktails). Lysates were denatured in 1 x sample loading buffer and resolved by SDS-PAGE, then the proteins were transferred to a polyvinylidene difluoride membrane. After blocking with 5% non-fat milk in Tris-buffered saline containing 0.1% Tween-20 (TBST), the membranes were incubated with different primary antibodies overnight at 4°C. The proteins were detected by chemiluminescence using an HRP substrate (GE Healthcare) after incubating the membrane with HRP-conjugated secondary antibody and washing with TBST. The images of western blotting were quantified by using ImageJ software (Wayne Rasband, NIH, Bethesda, MD).

### Lysosomal pH measurement

Briefly, HeLa cells were cultured in a black-walled, clear-bottomed 96-well plate (PerkinElmer, Inc. USA). After treatment of vehicle, RAP, CQ or 10 μM DAC and DAS for 24 h, the cells were washed and incubated with 5 μM of LysoSensor Yellow/Blue DND-160 (Invitrogen, USA) in complete DMEM without serum for 5 min at 37°C. Light emitted at 535 nm in response to excitation at 340 and 380 nm were determined and the ratio of them was calculated. The ratio was compared to the value of Control and CQ (a classic lysosomal pH neutralizer) treatment.

### *In vitro* lysosomal *V*-type ATPase activity

The assay was performed as previously described [29]. Briefly, HEK293 cells were incubated overnight with 35 μg/ml Dx-OG514. Cells were washed and incubated with serum-free DMEM for 2 h. 15 minutes prior to lysis, FCCP was added into the medium to a final concentration of 1 μM. Cells were scraped in fraction buffer (50 mM KCl, 90 mM K-Gluconate, 1 mM EGTA, 50 mM Glucose, 20 mM HEPES, protease inhibitor cocktail, pH=7.4) supplemented with 1 μM FCCP. After spraying with needle, cells were spun down at 10,000 rpm for 15 sec. at 4°C. Then, re-centrifuge the supernatant at max speed for another 20 minutes. The pellet was resuspended in pre-warmed fractionation buffer supplemented with 1% BSA, and split into several aliquots with DAC, DAS or BAF treatment for 30 min. Baseline fluorescence was measured at 530 nm upon 511 nm excitation in 96-well plate (PerkinElmer, Inc. USA) at 30s intervals for 5 min. 5 mM ATP and 5 mM MgCl_2_ were added into each well to activate the V-type ATPase, and continued recording the fluorescence for another 25 min. Active v-type ATPase caused lysosomal acidification and leaded a decrease in fluorescence emission of OG-514 over time.

### Statistical analysis

The significance of differences between two groups was determined by Student's *t* test, and the differences between multiple groups were determined by one way analysis of variance (ANOVA) with Turkey as post hoc tests. Calculations were performed with Prism software. Statistical significance was taken at p < 0.05.

## SUPPLEMENTARY MATERIALS FIGURE AND TABLE



## Abbreviations

DAC: Dauricine
DAS: daurisoline
CPT: campothecin
TXA2: thromboxane A2
AVs: autophagic vacuoles
RAP: rapamycin
CQ: chloroquine
BAF: Bafilomycin A1
Tet: tetrandrine
MEF: Mouse Embryo Fibroblast
