# Supplementary material for: Natural autophagy blockers, dauricine (DAC) and daurisoline (DAS), sensitize cancer cells to camptothecin-induced toxicity
Source: Oncotarget. 2017 Sep 8;8(44):77673–84. doi: 10.18632/oncotarget.20767 (PMC5652807; doi:10.18632/oncotarget.20767)
Supplement: Supplementary file 1 [file oncotarget-08-77673-s001.pdf]

Natural autophagy blockers, dauricine (DAC) and daurisoline (DAS), sensitize cancer cells to camptothecin-induced toxicity

SUPPLEMENTARY MATERIALS

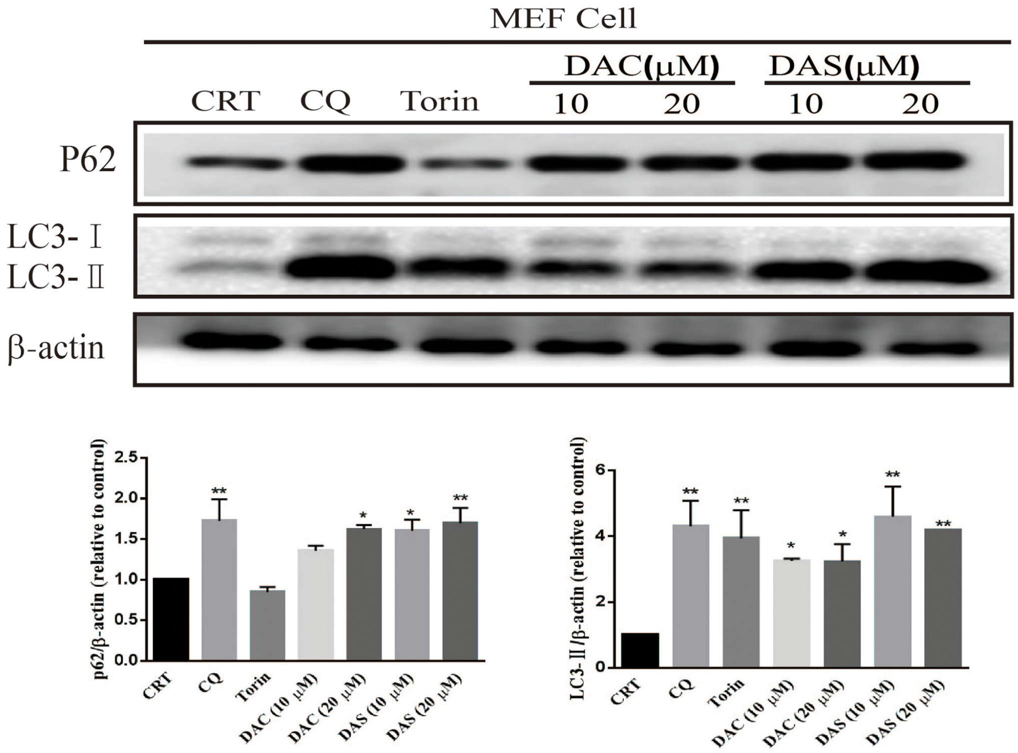

Supplementary Figure 1: P62 and LC3 expression on MEF cells after treatment of DAC and DAS. MEF cells were treated with DAC and DAS in different concentrations for 24 h, and the expression of P62 and LC3 were measured by western blotting. Relative intensity of p62 and LC3-IIwas calculated by Image J (*$P < 0.05$, **$P < 0.01$). Error bars are mean±SEM. One way ANOVA with Turkey as post hoc tests.

Supplementary Table 1: IC50 values of DAC and DAS on the HeLa, A549, and HCT-116 cancer cells

Cell type	Compound	IC50±SEM
HeLa	DAC	69.85±1.03
	DAS	74.75±1.03
A549	DAC	54.22±1.02
	DAS	50.54±1.02
HCT-116	DAC	94.44±1.04
	DAS	80.81±1.10

All cells are treated with DAC and DAS for 24 h.
